# Cancer Mortality and Deprivation in the Proximity of Polluting Industrial Facilities in an Industrial Region of Spain

**DOI:** 10.3390/ijerph17061860

**Published:** 2020-03-13

**Authors:** Vanessa Santos-Sánchez, Juan Antonio Córdoba-Doña, Javier García-Pérez, Antonio Escolar-Pujolar, Lucia Pozzi, Rebeca Ramis

**Affiliations:** 1 Department of Economics and Business, University of Sassari, Via Muroni 25, 07100 Sassari, Italy; lpozzi@uniss.it; 2 Department of Preventive Medicine and Public Health, Jerez University Hospital, Ronda de Circunvalación s/n, 11407 Jerez de la Frontera, Spain; jantonio.cordoba@juntadeandalucia.es; 3 Cancer and Environmental Epidemiology Unit, National Center for Epidemiology, Carlos III Institute of Health, Avenida Monforte de Lemos 5, 28029 Madrid, Spain; jgarcia@isciii.es (J.G.-P.); rramis@isciii.es (R.R.); 4 Center for Biomedical Research in Epidemiology and Public Health (CIBER Epidemiología y Salud Pública-CIBERESP), 28029 Madrid, Spain; 5 Consejería de Salud de la Junta de Andalucía, Cádiz, Avenida María Auxiliadora 2, 11009 Cádiz, Spain; antonio.escolar@uca.es

**Keywords:** cancer mortality, health inequalities, industrial pollution

## Abstract

Residential proximity to industrial facilities that release pollutants is a source of exposure to a high number of toxics, many of them known or suspected carcinogens. The objective of the study was to analyze the association between lung, larynx, bladder, and kidney cancer mortality and deprivation in areas proximate to polluting industrial facilities in Cadiz, a highly industrialized province in Spain. An ecological study at census tract level was carried out to estimate the mortality rates associated with deprivation and proximity to polluting industrial facilities (1–5 km) using the Besag–York–Mollié model. The results show a negative social gradient for lung and larynx cancers in males and greater risk of lung cancer was observed in the least deprived areas in females. These associations were found regardless the distance to industrial facilities. Increasing excess risk (relative risk; 95% credibility interval) of lung cancer for males (1.09; 1.02–1.16 at 5 km vs 1.24; 1.08–1.41 at 1 km) and bladder cancer for males (1.11; 1.01–1.22 at 5 km vs 1.32; 1.08–1.60 at 1 km) and females (1.32; 1.04–1.69 at 4 km vs 1.91; 1.28–2.86 at 1 km) was found as proximity to polluting industrial facilities increased. For kidney cancer, high risks were observed near such facilities for both sexes. Knowing the possible influence of industrial pollution and social inequalities over cancer risk allows the definition of policies aimed at reducing the risk.

## 1. Introduction

In Spain, cancer is the leading cause of death among males and the second leading cause among females [1]. Although the south-western part of the country is currently experiencing a decrease in cancer mortality rates, this region has historically seen higher rates than the rest of Spain, with this excess primarily affecting the Andalusian provinces of Cadiz, Huelva, and Seville [2]. Moreover, despite the decrease, cancer mortality rates in the province of Cadiz are higher than both Spanish and European estimates for all cancers, as well as for various specific cancers such as lung and laryngeal cancers in males or bladder and kidney cancers in females [3]. In 2017, the European age standardized rate for lung cancer in the province of Cadiz was 57.91 × 10^5^ in males and 12.53 × 10^5^ in females; for laryngeal cancer was 4.89 × 10^5^ in males and 0.40 × 10^5^ in females; for bladder cancer was 11.46 × 10^5^ in males and 1.13 × 10^5^ in females; for kidney cancer the rates were 7.15 × 10^5^ in males and 2.28 × 10^5^ in females [4].

Residential proximity to industrial facilities that release pollutants into the air and water is a source of exposure to a high number of toxics, many of them known or suspected carcinogens. In recent years, several studies have been carried out to assess the relationship between these emissions and the health of the local population. Cancer has been one of the most studied diseases in relation to residential proximity to industrial sites and, among them, cancers of the lung, larynx, bladder, and kidney have been positively associated with residential industrial exposure [5,6,7].

In 2002 and 2007, the European Commission developed the Integrated Pollution Prevention and Control (IPPC) register and the European Pollutant Release and Transfer Register (E-PRTR) [8]. These instruments obliged all facilities to declare all emissions above the designated thresholds of the E-PRTR, with this information then being used to create an inventory of geo-located facilities in Europe that have an environmental impact. This resource makes it possible to carry out analyses of the health impacts of residential proximity to polluting industrial facilities, such as the impact on cancer rates [9,10]. In 2001, in Europe, Spain was found to be the greatest emitter of almost one third of all E-PRTR-registered pollutant substances released into the air and among the three most contaminating countries for two-thirds of all such substances. In this context, the autonomous community of Andalusia, in the south of the country, stands out as the area that produces the largest volume of emissions of many of these pollutants [11].

The existing literature describes a well-documented relationship between the socioeconomic status of populations and cancer mortality, with mortality increasing in lower socioeconomic groups [12]. At present, there is thus a focus on whether the health risks associated with environmental pollution are equitably distributed among the population or are concentrated in areas of greatest deprivation [13]. Thus far, higher levels of pollution have been reported in more deprived areas [14].

The province of Cadiz, with a population of 1.2 million, is a highly industrialized area and is one of Spain’s most socioeconomically deprived regions, as reflected in its high rates of unemployment, poverty, and social exclusion [15]. In the 1960s, the Campo de Gibraltar was classified as a Preferred Industrial Location Zone, which provided industries settling in this territory with several benefits. Similarly, in the 1980s, the Bay of Cadiz was declared an Urgent Reindustrialization Zone. These initiatives envisioned that the region could constitute, along with Huelva and Seville, the three vertices of a triangle indispensable for the development of western Andalusia. In recent years, concern about the health effects of this wave of industrialization has motivated the investigation into the impact of environmental pollution on cancer over-mortality [16]. However, many of studies done to date have focused solely on the Campo de Gibraltar area and have thus neglected other areas within the affected province [17].

To address this gap in the research, the aims of this study were: (1) To analyze the association between cancer mortality, specifically, lung, larynx, bladder, and kidney cancer mortality, and deprivation and proximity to polluting industrial facilities by census tract in the province of Cadiz during the period 1992–2014; and (2) to analyze this mortality risk according to the categories of carcinogenic substances released by these industrial facilities.

## 2. Materials and Methods

An ecological study was carried out to analyze the association between cancer mortality, deprivation and proximity to industrial facilities at a census tract level (819 census tracts) in the province of Cadiz over the period 1992–2014.

### 2.1. Mortality Data

Census tract mortality data organized by cause, sex, age and year of death were obtained from the Andalusian Mortality Register. We studied cancer mortality for the following cancer types as described in the International Classification of Diseases (10th revision): Malignant neoplasm of the larynx (C32), malignant neoplasm of the lung (C33–C34), malignant neoplasm of the kidney (C64–C66) and malignant neoplasm of the bladder (C67).

The residential addresses of each observed case were geocoded using the Geocoder software (version 1.4.2, Junta de Andalucía, Sevilla, Spain) [18]. Those cases that were not automatically geocoded with this tool were manually geocoded using Google Earth and the directory provided by the National Statistics Institute [19]. Using these tools, we ultimately geocoded a total of 96.43% of the observed cases.

### 2.2. Socioeconomic Level

To determine the socioeconomic status of the observed cases, we used a deprivation index at the census tract level generated by [20], widely used in epidemiological studies in Andalusia [21,22]. Employing principal components analysis, a composite index is calculated based on the following variables: (i) Percentage of people with a low level of education; (ii) percentage of unemployed people; and (iii) percentage of unskilled workers. This combination of variables explains a high proportion of the variance (67%) and covers several domains of social class such as education and occupation, similarly to most deprivation indices. Each census tract is assigned a factorial score and classified according to the five levels of deprivation corresponding to the quintiles of the distribution of the factorial score. In the study, the census tracts found to be the least deprived, those at level 1, were considered to constitute the base level. Deprivation indices at small area level have proved to be remarkably stable over time as measures of socioeconomic status [23].

### 2.3. Industrial Pollution Exposure Data

The exposure to industrial pollution variable was estimated by calculating the distance from the geometric centroid of the census tract to each of the industrial facilities. Where the geometric centroid of the census tract was in an unpopulated or sparsely populated area, it was moved to the area with the highest population density.

**Figure 1:**
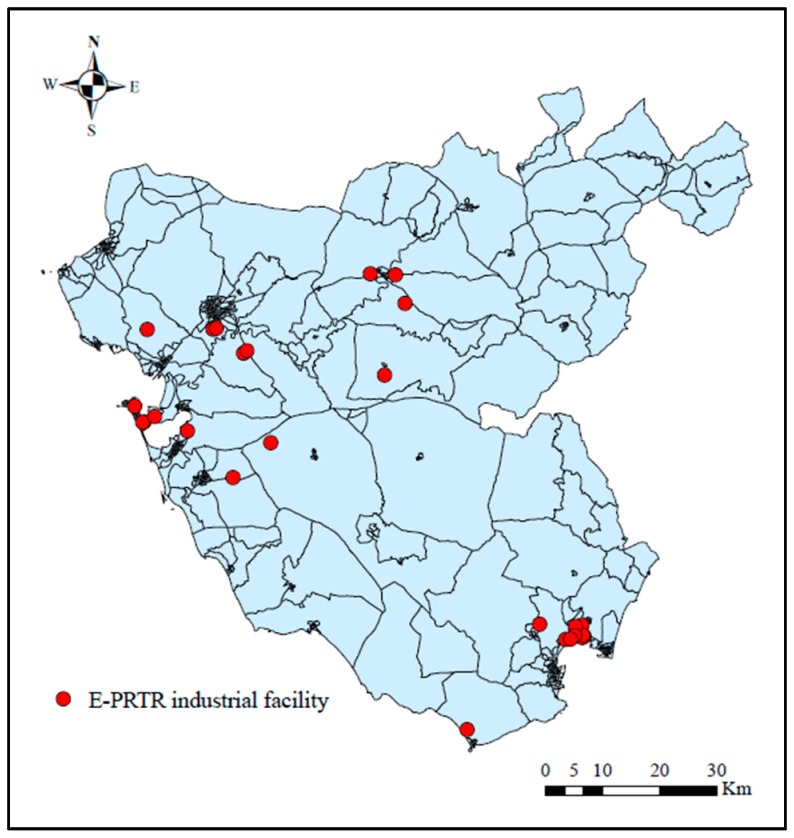
Locations of the 26 industrial facilities analyzed. E-PRTR—European Pollutant Release and Transfer Register.

The geographic coordinates of the industrial facilities were obtained from the Ministry of Agriculture, Food and the Environment’s 2010 E-PRTR and IPPC register. These coordinates were previously verified in response to the presence of errors in the initial coordinates provided in these registers [24]. Given that the latency period for the type of solid cancers under investigation is typically 10 years or more [25], the industrial facilities included in this study were those that started their operations before 1994, 10 years before the midpoint of the study period, and which released emissions into the air. Information on the year of commencement of industrial activity was obtained directly from the industrial facilities and the median year of the beginning of operation of the industries analyzed is 1972. Based on these criteria, a total of 26 facilities were included in this research, of which: Three facilities belong to the agro-food industrial group, three to the chemical, eight to the mineral, three are combustion facilities, three are production and processing of metals facilities, three are urban waste water treatment plants and three are shipbuilding facilities. The 26 installations analyzed are located within the province of Cadiz (Figure 1), since in the adjacent provinces all the installations are located at distances greater than 5 km from the centroid of any census tract of Cadiz.

### 2.4. Statistical Analysis

To calculate the number of expected cases, mortality rates for each cancer type and sex for the whole province was multiplied by the population of each census tract both by age group (17 5-year groups from 0 to 84 years and 1 group of those aged 85 years and over) and sex. The census tract, sex, and age data were obtained from the 2001 Census of the National Statistics Institute.

To assess the relationship between cancer mortality and residential proximity to polluting industrial facilities, two types of analyses were carried out:

(1) In the first phase, we created exposure variables to estimate the relative risks (RRs) of mortality associated with the distance to industrial facilities. The census tracts were stratified into the following levels: (a) Exposed area: Census tracts at distances less than 1, 2, 3, 4 and 5 km from a facility; (b) intermediate area: Census tracts with a facility at a distance greater than that considered as exposed in (a) but less than 5 km; (c) unexposed area: Census tracts with an IPPC-E-PRTR-registered facility more than 5 km away from their centroid. These unexposed census tracts were classified as the baseline group. In the analysis of exposure within 5 km, no intermediate exposure category was applied.

(2) The second phase focused on the relationship between cancer mortality and proximity to industrial facilities releasing substances classified by the International Agency for Research on Cancer (IARC) as carcinogenic to humans (Group 1) and possibly carcinogenic to humans (Group 2A). The facilities considered in the analysis emit the following substances belonging to Group 1: Arsenic and compounds, benzene, benzo(a)pyrene, cadmium and compounds, polycyclic aromatic hydrocarbons, nickel and compounds, polychlorinated dibenzo-p-dioxins + polychlorinated dibenzofurans (PCDD + PCDF), particulate matter (PM10) and trichloroethylene. Regarding Group 2A, the emissions were lead and compounds and tetrachloroethylene. For this phase, exposure variables were established for the industrial facilities that released Group 1 and Group 2A substances using the same methodology as that applied in phase 1 of the analysis.

To conduct the above analyses, smoothed RRs and their corresponding 95% credibility intervals were obtained using Poisson generalized linear-mixed regression models with two random effects, one capturing spatial dependence ($b_{i}$) and the other capturing heterogeneity or unstructured (non-spatial) overdispersion ($h_{i}$), as per the Besag–York–Mollié (BYM) model [26]. The model including the explanatory variables (socioeconomic level and exposure) was defined as follows:

$$O_{i}\sim Poisson\left(\mu_{i}\right),\;\mathrm{with}\;\mu_{i}{=E}_{i}\lambda_{i}\;\;{\log(\lambda }_{i}{)=\alpha Expos}_{i}{+\beta Soc}_{i}{+h}_{i}{+b}_{i}\overset{\;}{\Rightarrow }{\log(\mu }_{i})\;\;\;\;{=\log(E}_{i}{)=\alpha Expos}_{i}{+\beta Soc}_{i}{+h}_{i}{+b}_{i}\;i=\;1,\ldots ,\;819\;\mathrm{census}\;\mathrm{tracts}\;Expos=\;\mathrm{Exposure}\;\mathrm{variable}\;Soc=\;\mathrm{Socioeconomic}\;\mathrm{level}$$

where $O_{i}$ denotes the cancer mortality rates observed in the census tract *i*; $E_{i}$ are the expected cases; $\lambda_{i}$ is the RR and α and β are the estimators of the effects associated with each covariate. Exposure variables and socioeconomic level were considered fixed effects in the models. Integrated Nested Laplace Approximations (INLA) [27] were used to determine the Bayesian inference of subsequent marginal distributions via the R-INLA library available in the R statistical package [28].

## 3. Results

During the period 1992–2014, there were 10,728 reported deaths from lung cancer of which 9775 (91.12%) of the deceased were male and 953 (8.88%) were female; 1190 deaths from laryngeal cancer of which 1159 (97.39%) of the deceased were male and 31 (2.61%) were female; 2544 deaths from bladder cancer of which 2221 (87.30%) of the deceased were male and 323 (12.70%) were female; and 862 deaths from kidney cancer of which 568 (65.89%) of the deceased were male and 294 (34.11%) were female.

Table 1 and Table 2 illustrate the RRs of the model associated with the deprivation index for each sex and cancer type. This model responds to our first objective and applies the level 1 census tracts (those areas with the lowest levels of deprivation) as a baseline. It can be observed that the mortality RRs for each cancer type and sex show a similar distribution across all the distances analyzed. The results show a statistically significant negative social gradient among males for lung and laryngeal cancers, i.e., greater deprivation corresponds to a greater risk of death (Table 1). A positive social gradient (lower risk of death as deprivation increases) can be observed in relation to lung cancer among females from level 3 onwards (Table 2).

Table 3 shows the RRs of the exposed areas and their corresponding 95% credibility intervals obtained using the BYM model and considering the total number of industrial facilities in the E-PRTR and IPPC register. Statistically significant excess mortality risks, adjusted for deprivation index, were observed for lung cancer in males and for bladder and kidney cancers in both sexes. Results obtained for lung (RR = 1.09 at 5 km vs RR = 1.24 at 1 km) and bladder (RR = 1.11 at 5 km vs RR = 1.32 at 1 km) cancers in males and for bladder cancer in females (RR = 1.32 at 4 km vs RR = 1.91 at 1 km) are especially striking and reveal an increase in risk as proximity to industrial facilities increases.

Table 4 shows the RRs and 95% credibility intervals for facilities that release substances classified by the IARC as carcinogenic (Group 1) and possibly carcinogenic (Group 2A). Of the 26 facilities analyzed, 21 released substances belonging to Group 1 and 11 released substances belonging to Group 2A.

The mortality RR values obtained in the analysis involving Group 1 substances are very similar to those of the overall analysis for both sexes and all cancer types analyzed.

In the analysis involving Group 2A substances, among males, the figure for kidney cancer (RR = 1.19 at 5 km vs RR = 1.51 at 1 km) is striking. Moreover, significant excess risks of bladder cancer mortality in males (RR = 1.46 at 1 km) and females (RR = 1.72 at 1 km) were also observed.

## 4. Discussion

The results of our study reveal a significant social gradient associated with cancer mortality, but also a link between proximity to pollution industrial facilities and the studied cancer causes. For instance, for lung and laryngeal cancer mortality among males, the higher mortality risk was found in the most deprived census tracts. However, among females, the highest lung cancer mortality risk was observed in census tracts with the highest socioeconomic level. Nevertheless, the RRs remain almost the same at all distances analyzed, which indicates that distance to industrial facilities has no impact on these RRs. Regarding industrial exposure, the results obtained from the general analysis of the male population revealed an excess of mortality risk for lung, bladder and kidney cancers in the proximity of IPPC-E-PRTR-registered facilities after adjusting for socioeconomic level. Among females, there was an excess risk of bladder and kidney cancer mortality, which was more marked for bladder cancer. When stratified by Group 1 and Group 2A emissions, the results obtained for Group 1 emissions were similar to those returned by the analysis of all facilities most likely because all but five of the facilities analyzed emit carcinogenic substances belonging to this group. For Group 2A, significantly high RRs were obtained for bladder cancer in both sexes and kidney cancer in males in the proximity of polluting industrial facilities. The results obtained for each specific cancer type will be discussed below.

### 4.1. Lung Cancer

A clear social gradient can be observed for lung cancer mortality risk in males, while in females, risk decreases in the most deprived census tracts. This disparity could be explained by differences in lifestyle according to sex, like tobacco consumption, given that tobacco is responsible for 80% of cancers of this type. Tobacco use is more prevalent among males of lower socioeconomic status while the use of tobacco among females of higher socioeconomic status has been increasing since 1960 [29]. Although other intermediary determinants such as dietary factors, also related to socioeconomic status, have been associated to lung cancer mortality [30,31,32], we consider that tobacco consumption has the leading role on the association found between deprivation and mortality at all distances studied.

The results obtained for lung cancer in males show clear ascending risks as the distance to industrial facilities decreases, the highest risk being at distances of 1 km both in the general analysis and in the analysis of Group 1-emitting facilities. A relationship between lung cancer mortality and residential proximity to industrial facilities is identified in the existing literature [33]. One study by [6] analyzes lung cancer mortality in the populations residing in Spanish provinces near the combustion installations included in the EPER register (the previous industrial register replaced by the E-PRTR), adjusting for socio-demographic indicators. Of the provinces examined, Cadiz was found to have the highest standardized mortality ratios (SMRs) for lung cancer in males. This high mortality due to lung cancer has historically been associated with the prevalence of smoking among the population of Cadiz, as well as socioeconomic, environmental and occupational factors [2].

### 4.2. Laryngeal Cancer

A negative social gradient can be observed for laryngeal cancer mortality in males. These results reflect changes in lifestyles like a higher prevalence of smoking and/or alcohol consumption among males in lower socioeconomic classes. Among females, greater risk can be observed in level 1 (least deprived) census tracts in comparison with level 2, 3, and 4 areas possibly due to the greater prevalence of smoking and/or alcohol consumption among females of higher socioeconomic status [29,34]. At level 5 (most deprived), an excess mortality risk of approximately 20% is observed, which, as argued by [35], could be a consequence of the delay in the diagnosis of the disease among the most socioeconomically deprived females.

Regarding industrial proximity, the results suggest a not significant excess risk of mortality and thus support the findings of [36], which analyzed laryngeal cancer mortality in a highly industrialized Italian area. In the case of the female population, the lack of statistical significance could be due to the reduced sample size.

### 4.3. Bladder Cancer

For bladder cancer, the results concerning deprivation are inconclusive. Of most note is the moderate increase in the risk of mortality found among populations in census tracts experiencing intermediate levels of deprivation, a finding that is more marked among males. Again, these results suggest the presence of risk factors other than tobacco that could be affecting the population of Cadiz since there is a higher prevalence of smoking in lower socioeconomic groups, particularly among males.

Our study highlights the high risk for bladder cancer mortality seen among females near industrial facilities. A previous study [37], found that the bladder mortality rates for both sexes recorded in the province of Cadiz (as well as the provinces of Seville and Huelva, also a highly industrialized area) were among the highest in the country. This finding suggests the presence of a further determining factor in the area besides the high prevalence of smoking, the main risk factor for this type of cancer, such as environmental factors. In the existing literature, several authors have connected this cancer to both environmental and occupational factors. One of them is exposure to arsenic [38]. This metal can affect health through diet, water, or environmental exposure, but also, lifestyle factors, such as nutritional status, can influence arsenic metabolism, thus varying individual tolerance to arsenic toxicity [39,40]. Regarding occupational factors, a higher incidence of bladder cancer in metal workers, machinists, transport equipment operators, and miners in Western Europe was found [41], while other studies [42] found association between occupational exposure and risk for bladder cancer incidence and mortality.

### 4.4. Kidney Cancer

For kidney cancer, the results concerning deprivation are inconclusive although lower mortality risks for both sexes are observed at the level of greatest deprivation. The literature in this regard reports contradictory results, finding mortality risks to be both negatively and positively associated with high levels of deprivation [43,44].

Across all the industrial exposure analyses, an upward trend in the risk of kidney cancer mortality among males is found as the distance to industrial facilities decreases, with more significant values being recorded when examining facilities in terms of their emission of IARC-classified carcinogenic and possibly carcinogenic substances, which could be indicative of the effect of the aforementioned carcinogens on mortality. Certain occupational studies have found high mortality risks in populations exposed to these carcinogens, such as cadmium [45] and particulate matter [46].

## 5. Strengths and Limitations

One of the main strengths of the present study is the methodological approach applied. The use of hierarchical spatial models with explanatory variables reduces the risk of ecological fallacy [47] and ensures the geographical heterogeneity of mortality distribution. However, the estimates calculated using INLA requires a qualitative leap in the use of hierarchical models with explanatory variables [27].

Another strength lies in the use of the census tract as the spatial analysis unit for study, instead of the municipality, as the former tends to have a homogeneous size of between 1000 and 2500 inhabitants, which allows for the better determination of exposure at small distances and the better estimation of the effects of exposure on populations with similar socioeconomic and environmental characteristics.

Moreover, the large number of deaths recorded, due to the long study period, provides great statistical power, as well as partially compensating for the fact that using a small spatial unit reduces the overall number of deaths observed.

Other strengths that should be mentioned are the inclusion of those industrial facilities that started their operations prior to the middle of the study period and the precision of their location coordinates achieved through the exhaustive validation of these coordinates [24].

Nonetheless, our study also has limitations. One variable that may be acting as a confounder in our results is the level of tobacco consumption, an important risk factor for the cancers analyzed and for which there was no information at the census tract level. The separate analysis by sex using socioeconomic level as a covariate in the models represents an effort to minimize this effect since these factors are strongly associated with the different patterns of tobacco consumption among males and females. Likewise, another factor that could have influenced the different results obtained for the two sexes is occupational exposure, which, due to the lack of data, has been impossible for us to control.

Furthermore, although it has been partially compensated by moving the centroid of a census tract to the most populated area of that tract when it fell in an unpopulated or sparsely populated area, the use of the centroid to locate the entire population of the census tract may introduce bias [48]. However, this bias is reduced by the small extension of the census tracts since the potential health effects of pollution can extend over several kilometers.

Other potential limitations are the biases produced by possible population migrations, as well as wind, weather, and the characteristics of the terrain, which could influence the results when considering exposure to airborne and waterborne pollutants.

However, the above limitations only affect the identification of additional possible associations and do not invalidate the associations found in our study.

## 6. Conclusions

Our results support the hypothesis that, in the province of Cadiz, both socioeconomic level and residential proximity to facilities registered in the IPCC register and E-PRTR at a census tract level are independently related to an excess mortality risk for lung, larynx, bladder, and kidney cancers. Further research is needed to identify the possible risk factors responsible for the social inequalities found. Finally, knowing the possible influence of industrial pollution over cancer risk allows the definition of environmental and health policies aimed at reducing harmful exposures.

## Figures and Tables

**Table 1:** Relative risks (RRs) for deprivation index adjusted for distance of exposure for the male population.

Cause	DI	1 km			2 km			3 km			4 km			5 km		
		Exposed CT = 46			Exposed CT = 151			Exposed CT = 224			Exposed CT = 297			Exposed CT = 413		
		*n*	RR	95% CI	*n*	RR	95% CI	*n*	RR	95% CI	*n*	RR	95% CI	*n*	RR	95% CI
Lung cancer	1	174	1.00	-	390	1.00	-	542	1.00	-	694	1.00	-	1026	1.00	-
	2	74	**1.20**	1.09–1.32	372	**1.19**	1.08–1.32	662	**1.19**	1.08–1.31	876	**1.19**	1.07–1.31	1233	**1.19**	1.07–1.31
	3	65	**1.26**	1.14–1.39	418	**1.25**	1.13–1.38	610	**1.24**	1.13–1.37	803	**1.25**	1.13–1.37	1085	**1.25**	1.13–1.38
	4	125	**1.34**	1.21–1.47	448	**1.33**	1.20–1.46	536	**1.33**	1.21–1.47	704	**1.33**	1.20–1.47	934	**1.33**	1.21–1.47
	5	143	**1.37**	1.24–1.51	306	**1.36**	1.23–1.50	412	**1.36**	1.23–1.50	512	**1.36**	1.23–1.50	607	**1.37**	1.24–1.51
Laryngeal cancer	1	18	1.00	-	38	1.00	-	52	1.00	-	68	1.00	-	94	1.00	-
	2	5	**1.34**	1.06–1.70	37	**1.33**	1.05–1.68	74	**1.33**	1.05–1.68	95	**1.33**	1.05–1.68	129	**1.33**	1.05–1.68
	3	10	**1.70**	1.36–2.14	50	**1.69**	1.35–2.12	73	**1.69**	1.35–2.12	97	**1.69**	1.35–2.12	142	**1.69**	1.35–2.12
	4	20	**1.85**	1.47–2.31	53	**1.84**	1.47–2.31	73	**1.84**	1.47–2.30	101	**1.83**	1.46–2.29	121	**1.84**	1.47–2.30
	5	11	**2.09**	1.67–2.62	33	**2.09**	1.66–2.61	46	**2.08**	1.66–2.61	61	**2.07**	1.65–2.59	76	**2.08**	1.66–2.61
Bladder cancer	1	45	1.00	-	100	1.00	-	145	1.00	-	188	1.00	-	265	1.00	-
	2	16	1.12	0.96–1.30	98	1.11	0.95–1.29	170	1.10	0.95–1.28	215	1.10	0.95–1.28	282	1.10	0.95–1.28
	3	19	**1.20**	1.03–1.39	91	**1.18**	1.02–1.37	138	**1.18**	1.02–1.37	174	**1.18**	1.02–1.37	250	**1.18**	1.02–1.37
	4	32	**1.20**	1.03–1.39	99	**1.19**	1.02–1.38	127	**1.19**	1.02–1.38	168	**1.18**	1.02–1.38	221	**1.19**	1.02–1.38
	5	29	1.04	0.89–1.21	70	1.03	0.88–1.20	89	1.02	0.88–1.20	103	1.03	0.88–1.20	123	1.03	0.88–1.21
Kidney cancer	1	15	1.00	-	31	1.00	-	41	1.00	-	54	1.00	-	76	1.00	-
	2	5	0.99	0.76–1.28	17	0.97	0.74–1.25	38	0.96	0.74–1.24	56	0.95	0.74–1.24	76	0.96	0.74–1.24
	3	9	1.10	0.86–1.43	38	1.08	0.84–1.39	47	1.08	0.83–1.39	56	1.08	0.83–1.39	69	1.08	0.84–1.39
	4	7	0.96	0.73–1.25	23	0.94	0.72–1.22	30	0.94	0.72–1.23	37	0.94	0.72–1.22	53	0.94	0.72–1.23
	5	7	0.80	0.61–1.07	13	0.79	0.60–1.05	16	0.79	0.60–1.05	23	0.79	0.59–1.05	29	0.80	0.60–1.06

Models were estimated separately for each type of cancer and for the different cut-off points of the exposure variable. Statistically significant results are in bold. DI—deprivation index level; Exposed CT—number of census tracts at a distance less than that considered; *n*—number of observed cancer deaths; CI—credibility intervals.

**Table 2:** RRs for deprivation index adjusted for distance of exposure for the female population.

Cause	DI	1 km			2 km			3 km			4 km			5 km		
		Exposed CT = 46			Exposed CT = 151			Exposed CT = 224			Exposed CT = 297			Exposed CT = 413		
		*n*	RR	95% CI	*n*	RR	95% CI	*n*	RR	95% CI	*n*	RR	95% CI	*n*	RR	95% CI
Lung cancer	1	38	1.00	-	72	1.00	-	89	1.00	-	110	1.00	-	151	1.00	-
	2	3	0.91	0.75–1.11	30	0.89	0.74–1.08	63	0.89	0.73–1.08	93	0.89	0.73–1.07	124	0.89	0.73–1.07
	3	11	**0.81**	0.67–0.99	37	**0.80**	0.65–0.97	55	**0.79**	0.65–0.97	71	**0.80**	0.65–0.97	96	**0.80**	0.65–0.97
	4	10	**0.77**	0.63–0.94	36	**0.75**	0.62–0.92	44	**0.76**	0.62–0.92	61	**0.75**	0.62–0.92	76	**0.76**	0.62–0.92
	5	9	**0.63**	0.51–0.79	22	**0.63**	0.50–0.78	27	**0.63**	0.50–0.78	32	**0.62**	0.50–0.78	39	**0.63**	0.51–0.78
Laryngeal cancer	1	2	1.00	-	2	1.00	-	3	1.00	-	3	1.00	-	7	1.00	-
	2	0	0.79	0.27–2.28	1	0.78	0.27–2.25	2	0.80	0.28–2.31	3	0.81	0.28–2.34	4	0.80	0.28–2.31
	3	0	0.88	0.30–2.57	2	0.90	0.31–2.60	4	0.90	0.31–2.60	4	0.90	0.31–2.60	5	0.90	0.31–2.59
	4	0	0.81	0.26–2.49	1	0.83	0.27–2.55	1	0.81	0.26–2.51	2	0.83	0.27–2.55	3	0.81	0.26–2.51
	5	0	1.19	0.40–3.56	0	1.23	0.41–3.69	1	1.21	0.40–3.62	1	1.25	0.41–3.76	2	1.20	0.40–3.57
Bladder cancer	1	14	1.00	-	22	1.00	-	33	1.00	-	42	1.00	-	52	1.00	-
	2	3	0.84	0.58–1.19	19	0.80	0.56–1.14	30	0.79	0.56–1.13	34	0.79	0.55–1.13	40	0.80	0.56–1.13
	3	5	1.14	0.82–1.59	19	1.10	0.79–1.53	25	1.09	0.78–1.51	34	1.10	0.79–1.53	46	1.10	0.79–1.53
	4	3	1.03	0.73–1.45	13	1.01	0.72–1.41	16	1.01	0.72–1.41	24	1.01	0.72–1.41	35	1.01	0.72–1.42
	5	6	0.87	0.60–1.26	8	0.85	0.59–1.23	10	0.84	0.58–1.22	13	0.85	0.58–1.23	17	0.86	0.59–1.25
Kidney cancer	1	8	1.00	-	17	1.00	-	23	1.00	-	30	1.00	-	47	1.00	-
	2	5	1.18	0.85–1.65	16	1.15	0.83–1.60	25	1.15	0.83–1.60	34	1.15	0.83–1.60	48	1.15	0.83–1.60
	3	1	0.83	0.58–1.20	12	0.81	0.57–1.17	13	0.82	0.57–1.18	23	0.81	0.57–1.17	37	0.81	0.57–1.17
	4	7	0.86	0.60–1.23	18	0.84	0.59–1.21	22	0.85	0.59–1.22	28	0.85	0.59–1.22	31	0.85	0.59–1.22
	5	3	0.68	0.45–1.02	6	0.67	0.45–1.00	6	0.68	0.45–1.02	8	0.68	0.45–1.01	11	0.67	0.45–1.01

Models were estimated separately for each type of cancer and for the different cut-off points of the exposure variable. Statistically significant results are in bold.

**Table 3:** RRs of mortality from lung, laryngeal, bladder and kidney cancers adjusted for deprivation index in census tracts situated near polluting industrial facilities.

Cause	1 km			2 km			3 km			4 km			5 km		
	Exposed CT = 46			Exposed CT = 151			Exposed CT = 224			Exposed CT = 297			Exposed CT = 413		
	*n*	RR	95% CI	*n*	RR	95% CI	*n*	RR	95% CI	*n*	RR	95% CI	*n*	RR	95% CI
Lung cancer															
Men	581	**1.24**	1.08–1.41	1934	**1.14**	1.05–1.24	2762	**1.12**	1.04–1.20	3589	**1.11**	1.04–1.19	4885	**1.09**	1.02–1.16
Women	71	1.18	0.91–1.54	197	0.95	0.80–1.13	278	0.92	0.79–1.08	367	0.92	0.80–1.07	486	0.88	0.77–1.01
Laryngeal cancer															
Men	64	1.19	0.88–1.59	211	1.07	0.89–1.28	318	1.11	0.95–1.31	422	1.13	0.98–1.31	562	1.09	0.95–1.25
Women	2	1.67	0.36–7.78	6	1.48	0.53–4.14	11	1.89	0.78–4.55	13	1.72	0.73–4.02	21	1.99	0.91–4.37
Bladder cancer															
Men	141	**1.32**	1.08–1.60	458	**1.18**	1.05–1.34	669	**1.18**	1.06–1.31	848	**1.14**	1.03–1.26	1141	**1.11**	1.01–1.22
Women	31	**1.91**	1.28–2.86	81	**1.37**	1.03–1.82	114	**1.36**	1.05–1.76	147	**1.32**	1.04–1.69	190	1.24	0.98–1.57
Kidney cancer															
Men	43	**1.55**	1.12–2.17	122	1.23	0.99–1.54	172	1.18	0.97–1.44	226	1.18	0.98–1.42	303	1.14	0.95–1.35
Women	24	**1.59**	1.02–2.50	69	1.27	0.94–1.72	89	1.13	0.85–1.50	123	1.18	0.91–1.53	174	1.20	0.94–1.53

Models were estimated separately for each type of cancer and for the different cut-off points of the exposure variable. Statistically significant results are in bold.

**Table 4:** RRs of mortality from lung, laryngeal, bladder and kidney cancers adjusted for deprivation index in census tracts situated near polluting industrial facilities that release Groups 1 and 2A substances.

IARC Group 1 *															
**Cause**	**1 km**			**2 km**			**3 km**			**4 km**			**5 km**		
	**Exposed CT = 46**			**Exposed CT = 145**			**Exposed CT = 211**			**Exposed CT = 284**			**Exposed CT = 393**		
	***n***	**RR**	**95% CI**	***n***	**RR**	**95% CI**	***n***	**RR**	**95% CI**	***n***	**RR**	**95% CI**	***n***	**RR**	**95% CI**
Lung cancer															
Men	581	**1.23**	1.07–1.40	1865	**1.14**	1.05–1.23	2164	**1.11**	1.03–1.19	3420	**1.09**	1.02–1.17	4663	**1.08**	1.01–1.15
Women	71	1.20	0.92–1.56	192	0.97	0.81–1.15	269	0.96	0.82–1.12	357	0.95	0.82–1.09	471	0.90	0.79–1.03
Laryngeal cancer															
Men	64	1.20	0.89–1.61	204	1.09	0.91–1.31	304	1.14	0.97–1.34	413	**1.17**	1.01–1.35	542	1.12	0.98–1.28
Women	2	1.43	0.31–6.52	6	1.31	0.48–3.54	10	1.55	0.66–3.65	12	1.40	0.62–3.19	19	1.60	0.75–3.39
Bladder cancer															
Men	141	**1.31**	1.08–1.60	443	**1.18**	1.04–1.34	635	**1.18**	1.06–1.31	809	**1.13**	1.02–1.25	1088	**1.10**	1.00–1.21
Women	31	**1.89**	1.26–2.82	78	**1.35**	1.02–1.80	111	**1.37**	1.06–1.77	142	**1.30**	1.02–1.66	183	1.22	0.97–1.54
Kidney cancer															
Men	43	**1.59**	1.14–2.21	120	**1.28**	1.03–1.60	170	**1.27**	1.04–1.54	221	**1.24**	1.03–1.49	295	**1.19**	1.00–1.41
Women	24	1.53	0.98–2.40	67	1.22	0.90–1.65	85	1.09	0.82–1.45	117	1.11	0.86–1.44	164	1.13	0.89–1.44
IARC Group 2A **															
**Cause**	**1 km**			**2 km**			**3 km**			**4 km**			**5 km**		
	**Exposed CT = 26**			**Exposed CT = 87**			**Exposed CT = 158**			**Exposed CT = 240**			**Exposed CT = 370**		
	***n***	**RR**	**95% CI**	***n***	**RR**	**95% CI**	***n***	**RR**	**95% CI**	***n***	**RR**	**95% CI**	***n***	**RR**	**95% CI**
Lung cancer															
Men	318	1.11	0.93–1.32	1076	1.06	0.96–1.17	1872	1.04	0.96–1.13	2793	1.07	0.99–1.15	4355	**1.08**	1.01–1.15
Women	41	1.19	0.85–1.65	109	0.93	0.75–1.16	203	0.95	0.80–1.13	301	0.95	0.82–1.11	447	0.90	0.79–1.03
Laryngeal cancer															
Men	42	1.27	0.89–1.82	122	1.05	0.84–1.30	213	1.07	0.89–1.28	342	**1.16**	1.00–1.35	504	1.10	0.96–1.26
Women	2	2.60	0.57–11.85	3	1.17	0.32–4.18	7	1.52	0.58–3.95	10	1.48	0.62–3.51	19	1.80	0.85–3.83
Bladder cancer															
Men	92	**1.46**	1.15–1.86	257	1.14	0.98–1.32	461	**1.14**	1.01–1.29	659	1.10	0.99–1.23	1022	1.09	0.99–1.20
Women	15	**1.72**	1.00–2.95	35	1.14	0.78–1.66	80	**1.40**	1.05–1.85	116	**1.31**	1.01–1.68	174	1.22	0.97–1.54
Kidney cancer															
Men	25	**1.51**	1.00–2.30	79	**1.37**	1.06–1.77	137	**1.34**	1.09–1.66	194	**1.29**	1.07–1.56	279	**1.19**	1.00–1.41
Women	13	1.49	0.83–2.67	36	1.18	0.81–1.71	61	1.08	0.79–1.47	98	1.15	0.87–1.50	160	1.19	0.94–1.51

Models were estimated separately for each type of cancer and for the different cut-off points of the exposure variable. * Group 1 substances: arsenic and compounds, benzene, benzo(a)pyrene, cadmium and compounds, polycyclic aromatic hydrocarbons, nickel and compounds, polychlorinated dibenzo-p-dioxins + polychlorinated dibenzofurans (PCDD + PCDF), particulate matter (PM10) and trichloroethylene. ** Group 2A substances: lead and compounds and tetrachloroethylene. Statistically significant results are in bold. IARC— International Agency for Research on Cancer.
